# Knowledge about iodine requirements during pregnancy and breastfeeding among pregnant women living in Northern Ireland

**DOI:** 10.1186/s40795-019-0285-8

**Published:** 2019-03-12

**Authors:** Paul McMullan, Alyson Hunter, David McCance, Jayne V. Woodside, Karen Mullan

**Affiliations:** 1 Regional Centre for Endocrinology and Diabetes, Royal Victoria Hospital Belfast, Belfast, UK; 2 Centre for Public Health, School of Medicine, Dentistry and Biomedical Science, Queen’s University Belfast, Belfast, UK; 3 Royal Maternity Hospital Belfast, Belfast, UK

**Keywords:** Iodine, Knowledge, Nutrition, Pregnancy, Breastfeeding, Thyroid

## Abstract

**Background:**

Iodine is an essential micronutrient important for foetal nerve and brain development, especially in the early stages of pregnancy. The re-emergence of mild to moderate iodine deficiency has recently been reported in the United Kingdom (UK). The level of knowledge amongst pregnant women regarding iodine nutrition is poorly understood. The aim of this study was to determine the level of knowledge about iodine nutrition during pregnancy among pregnant women living in Northern Ireland (NI).

**Methods:**

A cross-sectional study in pregnant women was carried out in Royal Jubilee Maternity Hospital Belfast, from March to June 2015. Two hundred pregnant women were provided with a short questionnaire on iodine knowledge during routine clinic visits and comparisons were made across trimester and parity.

**Results:**

Only 20% of women were aware of the potentially increased iodine requirements during pregnancy and breast feeding; 45% were unable to identify any foods they thought would be iodine rich. The three main sources of dietary iodine in the UK are fish, dairy and eggs and 30, 9 and 15% correctly identified these as good sources respectively. When asked about whether they felt they had been given sufficient advice about folic acid and iodine in pregnancy, 90% felt this was so for folic acid, but only 5% for iodine.

**Conclusions:**

This study suggests that iodine knowledge among pregnant women living in NI is poor. In the absence of any iodine fortification programme, women in the UK may be vulnerable to iodine deficiency in pregnancy. At present they are poorly equipped to make positive dietary changes to meet their increasing iodine requirements during pregnancy and breastfeeding. Public health strategies should be considered to target this population group.

**Electronic supplementary material:**

The online version of this article (10.1186/s40795-019-0285-8) contains supplementary material, which is available to authorized users.

## Background

Iodine is an essential trace element required for production of thyroid hormones, and an increased supply of dietary iodine is of particular importance during pregnancy to compensate for foetal requirements [1]. Within the United Kingdom (UK) and Ireland, since the 1930s when iodine started to be added to cattle feed and successive UK governmemnts encouraged milk consumption in schoolchildren, iodine intakes were thought to be adequate. More recently, a survey reported iodine levels in teenage girls from the UK [2]. Results suggested that this group were mildly iodine deficient and that milk consumption positively correlated with iodine status as measured by urinary iodine concentration (UIC). Data from the Republic of Ireland (ROI) also indicated mild deficiency in the general population in the 1990s and, more recently, in pregnancy [3, 4]. Worldwide it is estimated that 1.9 billion people are affected by iodine deficiency [5].

Iodine is found in a range of foods, with the richest sources being fish and dairy products. Within Ireland and the UK, milk and dairy products tend to be the main sources of dietary iodine, [6, 7]. The UK and ROI have no formal iodine fortification programme in place, so that iodine status depends entirely on dietary intake from naturally occurring sources. This contrasts with many European countries and Australia which have a programme of salt iodization in place [8].

There are no UK specific recommendations on iodine supplementation or fortification during pregnancy, despite international guidelines (WHO 2007) [5]. The Scientific Advisory Committee on Nutrition (SACN) has advised no change to the status quo in the UK, pending further research [9].

Little is known if women themselves are aware of the importance of iodine in the perinatal period and if they are able to identify foods that are rich in iodine. In Northern Ireland (NI) ‘The Pregnancy Book’ is offered to all pregnant women [10]. Information is provided on folic acid, vitamin D, iron, vitamin C and calcium but does not mention iodine nor does it recommend iodine as supplement. The British Dietetics Association has produced a factsheet on iodine specifically for pregnant women, although awareness or uptake of this is uncertain [11]. There is a paucity of studies looking at iodine knowledge among pregnant women but those that are available point to clear gaps in knowledge. The aim of the study was to determine the level of knowledge about iodine nutrition during pregnancy among pregnant women living in Northern Ireland.

## Methods

### Subjects

Recruitment and data collection were carried out in the Royal Jubilee Maternity Hospital, Belfast Health and Social Care Trust between March and June 2015. Ethical approval was obtained from the Office of Research Ethics Committees Northern Ireland (Ref 14/NI/0047) and Governance approval from the Belfast Health and Social Care Trust (ref 13171KM-AS).

### Data collection

Two hundred questionnaires were distributed during routine antenatal clinics or education sessions by a member of the research team who explained the purpose of the questionnaire and offered a paper copy to those who provided consent.These clinics and visits occurred across trimesters. The numbers recruited were based on previous comparable questionnaire studies [12–14]. Consent was verbal and confirmed by completing and submitting the questionnaire. The questionnaire was self-completed without help and there were no inclusion /exclusion criteria beyond pregnancy. All participants were attending regular adult services and no children or vulnerable adults were approached. No personal information was collected from the questionnaire and data were fully anonymised. The questionnaire was set out in format in a similar way to previously reported questionnaires and checked for ease and clarity of instruction by 10 lay women (4 of whom were pregnant) [12, 13]. This is provided in the Additional file 1 section.

### Statistical analysis

Statistical analyses were conducted using the Statistical Package for the Social Sciences software (version 23.0; SPSS, Inc. USA) and significance set at *p* < 0.05. Non parametric testing was used in view of nominal and ordinal results from the questionnaire, with Mann-Whitney and Kruskall-Wallis tests used to compare results for 2 or ≥ 2 groups respectively. Data were presented using descriptive statistics and comparisons in responses among women at different stages and number of pregnancies were made.

## Results

A total of 183/200 (91.5%) completed and returned the questionnaire. Of these 59% were nulliparous. Most were in later stages of pregnancy with a breakdown of 4, 33 and 63% in 1st to 3rd trimester respectively. When asked to identify which gland iodine is needed for, 28% correctly identified the thyroid gland with 59% not sure. There was no significant difference in responses either between nulliparous and multiparous women or between trimesters. When asked to identify foods be rich in iodine nearly half of participants were not sure (Table 1).

**Table 1:** Responses to which foods are considered good sources of iodine

Food selected	*n*	%
Not sure	83	45
Seafood/fish^a^	55	30
Vegetables	44	24
Meat	42	23
Eggs^a^	28	15
Fruit	25	14
Bread	18	10
Dairy^a^	17	9
Salt	14	8
Poultry	10	6
Soya milk	9	5

^a^Sources rich in iodine in UK

The three main sources of dietary iodine in the UK are fish, dairy and eggs and 30, 9 and 15% correctly identified these as good sources respectively. Nulliparous women were less likely than multiparous women to identify seafood as a good source of iodine (22% vs 49%) (*p* = 0.006) but otherwise there were no differences between these two groups. There were no significant differences by trimester in the identification of the iodine rich foods.

Participants were asked “What happens to your iodine requirements during pregnancy and [breastfeeding]?” The majority were unsure for both pregnancy and breastfeeding while only ~ 20% answered correctly that iodine requirements increase during both periods (Table 2). There were no significant differences with parity or trimester in how women answered these questions. When asked of the effects of iodine deficiency, 5% of participants answered that they believed it to be harmful nearly half were unsure. No differences were seen between nulliparous and multiparous women or between trimesters. When asked “Can you have too much iodine in your diet?” the majority again were not sure especially among the nulliparous group (78% vs 59%) (*p* = 0.007) although there was no difference between trimesters (data not shown).

**Table 2:** Awareness of changes in iodine requirements during pregnancy and breast feeding and of harmful effects of deficient and excess iodine in the diet

Requirements?	*n*	%
During Pregnancy		
-Increase	39	22
-Decrease	29	16
-Same	3	2
-Not sure	108	60
During Lactation		
-Increase	37	21
-Decrease	16	9
-Same	3	2
-Not sure	123	69
Harmful effects?		
Deficiency		
-Yes	9	5
-No	89	50
-Not sure	82	45
Excess		
-Yes	5	3
-No	49	27
-Not sure	127	70

Participants graded the information given to them about folic acid, iron, calcium, vitamin D and iodine using a five point Likert scale (Table 3).

**Table 3:** Responses on a 5 point Likert scale for information adequacy during pregnancy regarding selected nutrients

Nutrient	*n*	%
Folic acid		
-Strongly disagree/Disagree	9	7
-Neither	6	4
-Agree/ Strongly agree	164	89
Iron		
-Strongly disagree/ Disagree	22	13
-Neither	19	11
-Agree/ Strongly agree	137	76
Vitamin D		
-Strongly disagree/ Disagree	39	23
-Neither	20	12
-Agree/ Strongly agree	118	65
Calcium		
-Strongly disagree/ Disagree	31	19
-Neither	24	14
-Agree/ Strongly agree	118	67
Iodine		
-Strongly disagree/ Disagree	129	76
-Neither	31	19
-Agree/ Strongly agree	7	5

The numbers of participants agreeing/strongly agreeing that adequate information was provided was highest for folic acid (89%) and lowest for iodine (5%). No differences were observed in response to this question when analysed by parity or trimester stage. Table 4 lists all the statements made in the comment section of the questionnaire.

**Table 4:** Responses for requests for any comments

	Comment
1	“Don’t know anything about iodine”
2	“Have pregnancy book which possibly provides more info on nutrients but I believe folic acid is promoted more than other nutrients obviously due to it being more vital in early pregnancy I believe”
3	“I did my own research into vitamin D but not know iodine was essential”
4	“I feel a lot of things are assumed and any info about nutrients has been by chance”
5	“I have never had iodine mentioned to me since I fell pregnant and no information”
6	“I have not been made aware of the significance of iodine during pregnancy”
7	“I haven’t read anything about iodine despite reading about diet in pregnancy”
8	“Iodine – not sure what this is”
9	“Iodine never discussed”
10	“Iodine was never explicitly mentioned but I did say I took pregnacare so I would guess that covers it”
11	“More info needed”
12	“Never had iodine mentioned at all”
13	“I thought iodine was bad for the baby’s development I didn’t think of it as something you should take”
14	“I have been told nothing about iodine during this pregnancy. I have gestational diabetes and this was not mentioned as an issue”
15	“Really don’t know much about this sorry”
16	“They ask loads of questions but give no feedback”

## Discussion

Our study findings are consistent with the two other studies available in the UK which show that knowledge around iodine in pregnant women is low. Table 5 compares recent studies in pregnant women and results are consistent over the last ten years. Knowledge was also deficient in a recent Norwegian cohort although a voluntary iodine fortification programme is in place which may mitigate against this to some extent [15]. A cohort from Australia also showed significant knowledge gaps among this vulnerable group but a mandatory fortification programme there is likely to counterbalance the lack of knowledge among this particular population [13].

**Table 5:** Iodine knowledge studies among pregnant women

Location	Years	No.	Cohort	Knowledge indexes	Scores	
Australia Charlton et al [13]	2007–8	139	Pregnant	Identify milk as a source of I	26%	
UK Combet et al [12]				Identify milk as a source of I	9%	
	2011–2	305	Pregnant	Awareness of I guidance	12%	
		721	Postpartum	Confidence to optimise I	28%	
UK Ref: [14] Bouga et al				Confidence to optimise I	29%	
	2015	48	Perinatal	Iodine awareness	23%	
Norway Gardweidner-Holme et al [15]					P	L
	2016	804	Pregnant	Identify milk as a source of I	29%	54%
		175	Lactating	Iodine knowledge score^a^	26%	45%
N Ireland				Identify dairy as source of I	9%	
	current	183	Pregnant	Adequacy of I information	5%	

^a^Self-reported on a scale (none: poor: low: medium: high); *P* Pregnant, *L* Lactating

Women report poor understanding of the importance of iodine in pregnancy, are inaccurate at identifying foods rich in iodine and are not confident about how to best optimise iodine intake in pregnancy. A similar study, but in non-pregnant women in the UK, was also consistent with these findings with 41% of participants unable to identify any health problem related to iodine deficiency [16].

Although knowledge regarding the importance of iodine is limited, motivation among women to make appropriate dietary changes in pregnancy is high as demonstrated in a qualitative paper on iodine in pregnancy conducted in 48 perinatal women in the UK [14]. The study also reported a desire by those in the perinatal period to have user friendly communication and involvement of health services to support these changes.

Although countries outside of the UK have shown similar low levels of knowledge, the UK population may be more vulnerable to possible iodine deficiency in pregnancy [13, 15]. The UK is one of twelve countries within the block of forty European countries which has no legislation for mandatory or voluntary iodine fortification [17]. We have recently reported in abstract form, iodine deficiency throughout all trimesters in 241 pregnant women from NI, which is separate cohort from that described in this paper [18]. This was despite 53% of women taking a pregnancy related supplement containing iodine.

It is interesting that broadly speaking, neither advanced trimester nor parity affected knowledge levels in our study and this would fit with a pattern of lack of accessible information for our pregnant women. The comments centre around a lack of information provided by health care providers and pregnancy literature provided. This contrasts markedly with adequacy of information regarding folic acid. The “Pregnancy Book” given to all pregnant women in NI discusses folic acid and advises supplementation of folic acid and vitamin D but fails to mention iodine. Bouga et al. points to community midwives as the main source of dietary advice in pregnancy in the UK but the advice received focussed on multivitamin supplements rather than food choices [14].

A recent Australian survey of 329 midwives reported that the majority rated both the importance of nutrition and the significance of their role in nutrition education as high [19]. Indeed the vast (93%) provided nutrition advice but their level of confidence in discussing nutrition issues ranged from low to moderate. Only half reported receiving nutrition education during their undergraduate or postgraduate careers. When asked about iodine requirements 80% gave incorrect responses.

Dietary knowledge about iodine has not always been shown to lead to significantly improved iodine status. O’Kane et al. reported that greater iodine knowledge scores were positively associated with a higher dietary intake of iodine in a non-pregnant population, as measured by a food frequency questionnaire (FFQ) [16]. However Garnweidner-Holme et al. reported no association between knowledge scores and iodine status in a pregnant/lactating cohort as demonstrated by the gold standard UIC [15]. They also reported that higher educated women and those who had received information about iodine had higher knowledge scores.

Public health strategies should be considered to increase awareness of the importance of iodine in pregnancy in this population group. Promotion of the BDA food fact sheet about iodine may be a good first step in this process although this may be an insufficient measure without a food fortification programme. Further work re iodine optimisation in this group is warranted.

This study has a number of limitations. Data on age, socio-economic status, education level and provision of any previous pregnancy nutritional education were not collected. No information was collected on those that did not complete the questionnaire or who did not want to take part. Those with poor nutritional knowledge may have declined completing this questionnaire potentially affecting results. All data were self-reported with the possibility of reporter bias but this pertains to all similar studies. Data on iodine knowledge among local midwives were not examined in parallel with this cohort and may be an interesting avenue as we endeavour to develop strategies to improve iodine nutrition in pregnancy.

## Conclusions

This study suggests that dietary iodine knowledge among pregnant women living in NI is poor. In the absence of any iodine fortification programme, women in the UK may be vulnerable to iodine deficiency in pregnancy. At present they are poorly equipped to make positive dietary changes to meet their increasing iodine requirements during pregnancy and breastfeeding.

## Additional file

Additional file 1: Questionnaire. Iodine Knowledge Questionnaire. Copy of questionnaire provided in study. (PDF 125 kb)

## Abbreviations

BDA: British Dietetic Association
NI: Northern Ireland
ROI: Republic of Ireland
SACN: Scientific Advisory Committee on Nutrition
SPSS: Statistical package for the Social Sciences
UIC: Urinary iodine concentration
UK: United Kingdom
WHO: World Health Organisation
