# Supplementary material for: Knowledge about iodine requirements during pregnancy and breastfeeding among pregnant women living in Northern Ireland
Source: BMC Nutr. 2019 Mar 12;5:24. doi: 10.1186/s40795-019-0285-8 (PMC7050819; doi:10.1186/s40795-019-0285-8)
Supplement: Supplementary file 1 — Questionnaire. Iodine Knowledge Questionnaire. Copy of questionnaire provided in study. (PDF 125 kb) [file 40795_2019_285_MOESM1_ESM.pdf]

Belfast Health and Social Care Trust

Iodine knowledge questionnaire

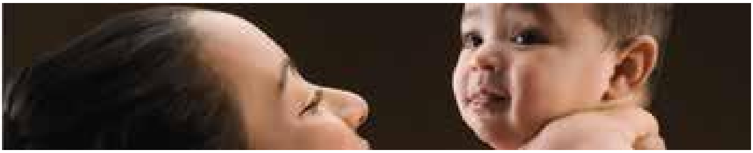

This questionnaire is designed to tell us about a nutrient in your diet called iodine. We are currently undertaking a study looking at this in detail but we are keen to find out what pregnant women know about this nutrient.

Your answers are confidential and your personal details are not requested in this questionnaire.

Please answer every question, if you are unsure about how to answer, please do the best you can.

CONTACT INFORMATION

Dr Paul McMullan
Regional Centre for Endocrinology and Diabetes
Royal Victoria Hospital
Grosvenor Road
Belfast BT12 6BA

Phone: 02890 247026 (a message can be left on the answer machine at all times)
Mobile: 07872580589
Email: paul.mcmullan@belfasttrust.hscni.net

1. **Is this your first pregnancy?** Yes ☐ No ☐

2. **How many <u>weeks</u> pregnant are you?**

   <12 ☐ **13-28** ☐ **>29** ☐

3. **Which foods are considered good sources of iodine in the U.K diet?**

Bread	☐	Poultry	☐	Meat	☐
Fruit	☐	Seafood/fish	☐	Salt	☐
Dairy	☐	Soya milk	☐	Eggs	☐
Vegetables	☐	Not sure	☐		

4. **Which gland in the body needs this nutrient called iodine to produce essential hormones?**

   Adrenal ☐ Thyroid ☐ Ovaries ☐

   not sure ☐ other ☐ (if other, please state)-----------

5. **What happens to your iodine requirements during pregnancy?**

   Increase ☐ Decrease ☐ Stays the same ☐

   Not sure ☐

6. **What happens to your iodine requirements if you breast feed?**

   Increase ☐ Decrease ☐ Stays the same ☐

   Not sure ☐

7. Are you aware of any potentially harmful effects from a lack of adequate iodine in your diet?

Yes ☐ No ☐ Not sure ☐

If yes, what potential harmful effects are you aware of? ______________________________

8. Can you have too much iodine in your diet?

Yes ☐ No ☐ Not sure ☐

9. After becoming pregnant, do you feel you were given enough information about the following nutrients? (mark with X)

	Strongly disagree	Disagree	Neither	Agree	strongly agree
Calcium	☐	☐	☐	☐	☐
Folic Acid	☐	☐	☐	☐	☐
Iodine	☐	☐	☐	☐	☐
Iron	☐	☐	☐	☐	☐
Vitamin D	☐	☐	☐	☐	☐

Any other comments?
